# Putative cause of seizure-induced cognitive alterations: The oscillatory reconfiguration of seizure network

**DOI:** 10.3389/fnins.2023.1126875

**Published:** 2023-01-19

**Authors:** Denggui Fan, Lixue Qi, Zecheng Yang, Guoming Luan, Qingyun Wang

**Affiliations:** ^1^School of Mathematics and Physics, University of Science and Technology Beijing, Beijing, China; ^2^Epilepsy Center, Sanbo Brain Hospital, Capital Medical University, Beijing, China; ^3^Department of Dynamics and Control, Beihang University, Beijing, China

**Keywords:** focal epilepsy, effective brain network, oscillatory reorganization, seizures transitions, cognitive impairment

## Abstract

**Introduction:**

The dynamic reconfiguration of network oscillations is connected with cognitive processes. Changes in how neural networks and signaling pathways work are crucial to how epilepsy and related conditions develop. Specifically, there is evidence that prolonged or recurrent seizures may induce or exacerbate cognitive impairment. However, it still needs to be determined how the seizure brain configures its functional structure to shape the battle of strong local oscillations vs. slow global oscillations in the network to impair cognitive function.

**Methods:**

In this paper, we aim to deduce the network mechanisms underlying seizure-induced cognitive impairment by comparing the evolution of strong local oscillations with slow global oscillations and their link to the resting state of healthy controls. Here, we construct a dynamically efficient network of pathological seizures by calculating the synchrony and directionality of information flow between nine patients’ SEEG signals. Then, using a pattern-based method, we found hierarchical modules in the brain’s functional network and measured the functional balance between the network’s local strong and slow global oscillations.

**Results and discussion:**

According to the findings, a tremendous rise in strong local oscillations during seizures and an increase in slow global oscillations after seizures corresponded to the initiation and recovery of cognitive impairment. Specifically, during the interictal period, local strong and slow global oscillations are in metastable balance, which is the same as a normal cognitive process and can be switched easily. During the pre-ictal period, the two show a bimodal pattern of separate peaks that cannot be easily switched, and some flexibility is lost. During the seizure period, a single-peak pattern with negative peaks is showcased, and the network eventually transitions to a very intense strong local oscillation state. These results shed light on the mechanism behind network oscillations in epilepsy-induced cognitive impairment. On the other hand, the differential (similarity) of oscillatory reorganization between the local (non) epileptogenic network and the global network may be an emergency protective mechanism of the brain, preventing the spread of pathological information flow to more healthy brain regions.

## 1. Introduction

Oscillations arise from neuronal interactions that promote communication and information processing between regions of a functional neural network (Engel et al., 2001; Buzsaki and Draguhn, 2004; Wang et al., 2020; Földi et al., 2021). Recent studies have shown that network oscillations temporally link neurons and enhance synaptic plasticity, supporting long-term information consolidation and is a cognitive process necessary for learning and memory (Goltsev et al., 2013; Holmes, 2015; Sadaghiani and Kleinschmidt, 2016). Specifically, tiny timing mistakes in neuronal or oscillatory activity may be magnified in more extensive networks, resulting in cognitive impairment (Buzsáki, 2007). This shows that using network oscillations to investigate cognitive impairment is a viable treatment strategy. Many studies are becoming more interested in the impact of network oscillations on cognitive impairment, focusing on specific frequencies and amplitudes of oscillation (Wilke et al., 2011; Ibrahim et al., 2012; Guo et al., 2018). However, the mechanism through which oscillatory reorganization influences cognitive impairment remains unknown.

In the present study, we surveyed the mechanisms through which network oscillations contribute to seizure-induced cognitive impairment. As a starting point, we investigated the findings of prior studies on the topic. Previous research has shown a causal relationship between the pathophysiological mechanisms that cause seizures and the biology of cognitive impairment, with oscillations acting as one of the essential links (Jensen et al., 2007; Guo et al., 2018). Gamma oscillations are closely associated with sustained learning and memory functions, and oscillation abnormalities may result in cognitive deficits (Ibrahim et al., 2012; Guo et al., 2018). Cognitive memory may be disrupted by seizures caused by intermittent oscillations (Binnie and Marston, 1992; Holmes and Lenck-Santini, 2006; Lévesque et al., 2018). In epileptic patients, abnormalities in brain connection produced by oscillations and impaired temporal coding affect cognition (Holmes, 2015). In patients with temporal lobe epilepsy, seizure-evoked circuits are localized in memory-supporting brain regions, and these regions generate essential physiological high-frequency oscillations required for memory processing (Axmacher et al., 2008; Ewell et al., 2019). However, most research has been limited to specific oscillations or epileptic disorders. Consequently, little is known about the systematic contributions of oscillatory reorganization to cognitive impairment. In addition, several studies have shown that strong local oscillations are more widespread in neuronal networks, with the local connection of neurons limiting their extension (Abela et al., 2014). Slow global oscillations reflect the integration of neuronal activity across regions of the brain throughout sensory or cognitive processes. However, recording technologies have limited investigations of precise mechanisms (Sheybani et al., 2019). There is no evidence that the encoded form of slow global oscillations applies to distributed networks in epileptic diseases. As a result, future research should examine the effects of oscillatory reorganization on seizures and cognitive impairment *via* the joint of strong local oscillations and slow global oscillations.

Moreover, there may be a mutually suppressive relationship between strong local oscillations and slow global oscillations. High-frequency oscillations are connected with local neuronal interactions, whereas slow rhythmic oscillations at lower frequencies are more crucial for the long-distance integration of large-scale networks (Von Stein and Sarnthein, 2000; Donner and Siegel, 2011; Goltsev et al., 2013; Ibrahim et al., 2013). When α oscillation energy rises in a specific brain region, neurogenesis in that region generally decreases. However, oscillatory reorganization’s precise effect on cognitive impairment has yet to be investigated, and many fundamental concerns remain unanswered. We have yet to determine, for instance, what change patterns occur in strong local oscillations under the effect of seizures, nor whether strong local oscillations play a role in suppressing slow global oscillations. In this research, we aimed to answer these crucial questions.

Furthermore, brain function does not originate from isolated brain regions but through interactions in large-scale networks (van Diessen et al., 2014), which seem essential for both physiological and pathological conditions. On the one hand, such connections might propagate seizures; or instance, interconnected focal regions may have suffered damage due to peaked wave dissemination, which often results in widespread cognitive impairment (Harkin et al., 2007). Alternatively, other research shows that the brain may have a mechanism to protect healthy brain regions from seizures, which may cause permanent damage. For instance, seizure cessation is characterized by somewhat uniform oscillatory suppression (Truccolo et al., 2014). Intermittent epileptiform discharges have remote inhibitory effects on cognition (Shamshiri et al., 2017; Ung et al., 2017; Guo et al., 2018; Watson, 2018). However, the mechanisms and pathophysiology that control particular functions have yet to be entirely understood. It is uncertain if the resting or task state of the healthy brain exists or has been altered in epileptic patients.

In this research, the pathophysiological mechanisms underlying epilepsy-induced cognitive impairment may be associated with the oscillatory reorganization of functional networks. We focus specifically on the mutually inhibiting effect of strong local oscillations and slow global oscillations. The experiment was based on a quantitative analysis of the synchronization and directionality of information flow between the SEEG signals of nine patients with pathological epilepsy to create a dynamic network. Previous research has only used indicators of complex networks, focusing on particular global or local connection changes. In contrast, we utilize methods based on characteristic mode to identify hierarchical modules and quantify strong local oscillations and slow global oscillations in the network to examine dynamic networks comprehensively. To provide new information, we identify the particular variation rules of strong local oscillations versus slow global oscillations during various seizure phases. These models and findings help characterize an unrecognized dynamic network oscillatory reorganization mechanism in cognitive impairment induced by epilepsy.

## 2. Materials and methods

### 2.1. Data preparation

In this research, nearly 100 channels (electrodes) of SEEG data from nine patients hospitalized with refractory focal epilepsy at the Sanbo Brain Hospital of Capital Medical University were visually analyzed and extensively exploited to validate our results independently. The Ethics Committee approved the SMBC of Capital Medical University’s study technique, and a negotiated informed special dispensation was prepared for all participants. This information cannot be shared with the general public due to hospital regulations. Furthermore, the sampling frequency was set at 512 Hz and the average monitoring time was 1 week. Interictal, preictal, seizure, and post-ictal periods were analyzed to determine the network features of people with epilepsy. For each patient, we devised criteria for categorizing the various seizure stages, as indicated in Table 1. These criteria were based on the clinical seizures recognized by physicians.

**TABLE 1:** Clinical patient characteristics.

Patient	Age (years)	Duration (years)	Side	Electrodes/contacts	Recorded seizures	Pathology
1	17	12	R	15/124	4	FCD Ia
2	9	5	L	11/116	6	FCD Ib
3	4	7/12	R	13/122	9	FCD IIa
4	7	1	L	10/120	101	FCD IIb + FCD Ic
5	5	3	R	10/108	2	FCD Ib
6	16	3	L and R	15/119	4	FCD Ib + FCD IIb
7	7	5	L	13/116	1	FCD Ib
8	27	12	L	8/108	5	HS.
9	15	9	R	8/117	17	FCD Ib + GMH

FCD, cortical dysplasia; HS, hippocampal sclerosis; GMH, ectopic gray matter.

The data for the control group in this study were collected from the EEG Motion/Image Dataset public dataset (Schalk et al., 2004), which includes over 1,500 1- and 2-min EEG recordings from 109 volunteers with 64 electrodes. The volunteers were required to perform a variety of motion and visual tasks. We selected nine data points from healthy individuals from which we extracted segments of resting or task-state EEG to compare with patients.

### 2.2. Statistic method for epileptic effective brain network

Usually, Pearson correlation coefficients are employed to establish functional networks for analyzing resting-state brain network alterations in healthy individuals. However, compared to FMRI data, the SEEG data employed in this work includes more complex dynamic changes in transients due to its temporal and spatial features. Consequently, this research establishes an effective network based on the statistical method proposed in Quiroga et al. (2002), which may improve the assessment of the information flow in the brain network of patients with epilepsy.

We build a dynamic and efficient network by estimating the synchronization and directionality of developing information flow to represent pathological epilepsy. Therefore, it is essential to define events for time series. Here, the events are defined as the local maximums in the time series, i.e.,


(1) $$\left\{ \begin{array}{c} x_{t_{k}}>x_{t_{k}+m},m=-M+1,\ldots ,-1,1,\ldots ,M-1\\ x_{t_{k}}>x_{t_{k}\pm M}+h\; \end{array}\right.$$

where *t*_*k*_ is the event occurrence time, *M* = 10*h* = 30 are the two control parameters shaping the event.

Let the event sequences of time series *x*^1^ (*n*) and *x*^2^ (*n*) (total step length *L*) be $t_{r}^{1}\left(r=1,\ldots ,m_{1}\right)$ and $t_{s}^{2}\left(s=1,\ldots ,m_{2}\right)$, where *m*_1_ (≪ *L*) and *m*_2_ (≪ *L*) are the total number of event occurrences in *x*^1^ (*n*) and *x*^2^ (*n*), respectively. The time difference between event occurrences in various time series is then used to determine the causality and synchronization of events. In the time delay scale τ, if an event is recorded in the sequence *x*^1^ (*n*) immediately after an event occurs in the sequence *x*^2^ (*n*), then it is considered that the intensity of the causal effect of *x*^1^ (*n*) on *x*^2^ (*n*) will be enhanced by one step. Conversely, *x*^2^ (*n*) is one step more causality for *x*^1^ (*n*). In addition, regardless of the sequence of events in *x*^1^ (*n*) and *x*^2^ (*n*), as long as two related events are observed to occur close enough, they are considered a simultaneous occurrence, and the amount of synchronization is correspondingly increased by one step.


(2) $$e^{\tau }\,\left(x^{1}|x^{2}\right)=\sum_{r=1}^{m_{1}}\sum_{s=1}^{m_{2}}E_{r\,s}^{\tau },E_{r\,s}^{\tau }=\left\{ \begin{array}{c} 1,0<t_{r}^{1}-t_{s}^{2}\leq \tau \\ \frac{1}{2},t_{r}^{1}=t_{s}^{2}\;\\ 0,o\,t\,h\,e\,r\,s\; \end{array}\right.$$

Remember sequence *x*^2^ (*n*) for *x*^1^ (*n*) event-causal *e*^τ^(*x*^1^|*x*^2^), the size of representative sequence *x*^2^ (*n*) for *x*^1^ (*n*) the causal role of strength. The global time delay,


$$\tau =\min_{r=1,\ldots \,m_{1},s=1,\ldots ,m_{2}}\left\{\tau_{r\,s}\right\}\,,$$

(3) $$\tau_{r\,s}=\frac{1}{2}\,\min\left\{t_{r+1}^{1}-t_{r}^{1},t_{r}^{1}-t_{r-1}^{1},t_{s+1}^{2}-t_{s}^{2},t_{s}^{2}-t_{s-1}^{2}\right\}$$

is used to limit the smallest time interval between two adjacent events, where τ_*rs*_ is the local time delay for each pair of adjacent (temporally closest) events (*r*, *s*), $E_{r\,s}^{\tau }=1/2\,$ is such set to prevent from double counting for the two simultaneous events. On the contrary, *e*^τ^(*x*^2^|*x*^1^) quantifies the causality from *x*^1^ (*n*) to *x*^2^ (*n*).

Based on *e*^τ^(*x*^1^|*x*^2^) and *e*^τ^(*x*^2^|*x*^1^), we then define the degree of simultaneity *Q*^τ^ and causality *q*^τ^ for *x*^1^(*n*) and *x*^2^(*n*) as follows:


(4) $$Q^{\tau }=\frac{e^{\tau }\,\left(x^{2}|x^{1}\right)+e^{\tau }\,\left(x^{1}|x^{2}\right)}{\sqrt{m_{1}\cdot m_{2}}},q^{\tau }=\frac{e^{\tau }\,\left(x^{2}|x^{1}\right)-e^{\tau }\,\left(x^{1}|x^{2}\right)}{\sqrt{m_{1}\cdot m_{2}}}$$

Both are normalized to 0 ≤ *Q*^τ^ ≤ 1, −1 ≤ *q*^τ^ ≤ 1. *Q*^τ^ = 1 when and only when all events in both signals occur together. *q*^τ^ = 1 when and only when all events from *x*^1^(*n*) act for all events from *x*^2^ (*n*).

Second, to examine the evolution trend of synchronization and causality between the two sequences over time, *Q*^τ^ and *q*^τ^ are evaluated for each time step, while their cumulative consequences are investigated over time. We define *q*^τ^(*n*) as Equation(5),


$$\left\{ \begin{array}{c} q^{\tau }\,\left(n\right)=e_{n}^{\tau }\,\left(x^{2}|x^{1}\right)-e_{n}^{\tau }\,\left(x^{1}|x^{2}\right)\\ Q^{\tau }\,\left(n\right)=e_{n}^{\tau }\,\left(x^{2}|x^{1}\right)+e_{n}^{\tau }\,\left(x^{1}|x^{2}\right) \end{array}\right.$$

(5) $$\,e_{n}^{\tau }\,\left(x^{1}|x^{2}\right)=\sum_{r=1}^{m_{1}}\sum_{s=1}^{m_{2}}E_{r\,s}^{\tau }\,\text{Θ}\,\left(\text{n}-{\text{t}}_{\text{r}}^{1}\right)\;$$

where *n* = 1, 2,…,*L* is the time point within the series, and Θ is the step function, i.e., Θ(x) = 1 when *x* > 0 and Θ(x) = 0 when *x* ≤ 0. *q*^τ^(*n*) could be thought of as a random sequence; when the event in *x*^1^ causes the event in *x*^2^ to occur, it increases by one step, and vice versa, it diminishes by one step. The synchronization of progressive events is defined by *Q*^τ^(*n*) in Equation (5), where *Q*^τ^(*n*) improves by one step if a pair of events in *x*^1^ and *x*^2^ occur within the period τ and remains nearly constant otherwise.

The rate of change of synchronization at time point n is calculated by averaging the synchronization expansion throughout Δ*n* steps (Δ*n* = 5120 in the current calculation) using *dQ*^τ^(*n*) in Equation (6),


(6) $$d\,Q^{\tau }\,\left(n\right)=\frac{Q\,\left(n\right)-Q\,\left(n-\Delta \,n\right)}{\sqrt{\Delta \,n_{1}\cdot \Delta \,n_{2}}},d\,q^{\tau }\,\left(n\right)=\frac{q\,\left(n\right)-q\,\left(n-\Delta \,n\right)}{\sqrt{\Delta \,n_{1}\cdot \Delta \,n_{2}}}$$

where Δ*n*_1_ and Δ*n*_2_ correspond to the number of events in *x*^1^ and *x*^2^ in the band [*n*−Δ*n*, *n*], respectively. Similarly, we might define the rate of change of the causal level at time point *n* as choosing to follow *dq*^τ^ (*n*) in Equation (6), where *dQ*^τ^ (*n*) > 0 and *dq*^τ^ (*n*) > 0 represent a positive increment of synchronization and causality within Δ*n* steps, respectively, and <0 represents a negative escalation within Δ*n* steps.

Specifically, the synchronization growth rate *dQ*^τ^ (*n*) and the causal level change rate *dq*^τ^ (*n*) at time point *n* are determined for any two nodes *i* and *j* in the network whose relevant time series are *x^i^* and *x^j^*. The magnitude of the weighted directed action of node *i* on node *j* at time point *n* is represented by *a*_*ij*_(*n*), which also contains data on the synchronization level between the two nodes, as described by Equation (7),


(7) $$a_{i\,j}\,\left(n\right)=\left\{ \begin{array}{c} \gamma \cdot d\,q_{i\,j}^{\tau }\,\left(n\right)\cdot d\,Q_{i\,j}^{\tau }\,\left(n\right),d\,q_{i\,j}^{\tau }\,\left(n\right)>0\\ 0,d\,q_{i\,j}^{\tau }\,\left(n\right)\leq 0 \end{array}\right.$$

where the amplification factor γ = 1000 is considered in this research. Furthermore, it is assumed that there are no self-connecting rings in the network, i.e., *a*_*ii*_ (*n*) = 0, *i* = 1, 2,…,*n*_0_, where *n*_*0*_ is the maximum number of nodes in the network.

### 2.3. Hierarchical modular division of functional connection matrix

**FIGURE 1:**
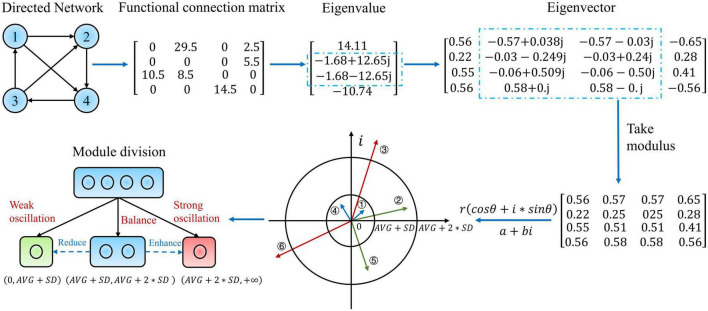
Example illustration and detailed description of the eigenmode analysis method. This diagram demonstrates a weighted directed network with four nodes. This network is then assigned a functional connectivity matrix corresponding to the hundred-dimensional matrix acquired from the real epilepsy causal effects network. The eigenvalues and eigenvectors of this matrix are then determined. The eigenvectors are then retrieved in modal length, and their interpretation in the complex plane is provided. The module is subdivided into three submodules based on the modal lengths of the eigenvectors, and the subfigure at the bottom left depicts the precise meaning and classification links of the hierarchical modularity method to oscillatory dynamic reorganization.

Wang Rong et al. (Wang et al., 2021) proposed that the activation and combination of various structural modal interactions in the eigenmodes lead to generating multiple dynamical modes in the system by nature. Moreover, in this study, to discover the dynamical oscillation reorganization law of the directed weighted network, we propose a new hierarchical eigenmode analysis method that includes examining complex numbers. In particular, the directed weighted network and functional connection matrix in Figure 1 is an epitome of the causal effects network calculation results. Since the functional connection matrix is asymmetric, the resulting eigenvalues and eigenvectors must include complex conjugate numbers. It is well known that the eigenvalues on the exact number field domain represent the magnitude of the stretching transformation, whereas the eigenvectors represent the direction. In comparison, the complex domain adds a rotational transformation. Under the transformation of polar coordinates, *a+bi* becomes *r*(*cos*θ + i_*_sinθ), where $r=\sqrt{a^{2}+b^{2}}$ represents the stretching quantity, and $\theta =a\,r\,c\,t\,a\,n\,\frac{b}{a}$ represents the rotation. Additionally, the complex eigenvector component *m* + *ni* is multiplied with the corresponding eigenvalue *a* + *bi*, representing the real rotation of the basis vector φ and the stretching transform *q* (Hitzer, 2002):


(a+b⁢i)⁢(m+n⁢i)=r⁢(c⁢o⁢s⁢θ+i*s⁢i⁢n⁢θ)*q⁢(c⁢o⁢s⁢φ+i*s⁢i⁢n⁢φ)=

r⁢q⁢[c⁢o⁢s⁢θ⁢c⁢o⁢s⁢φ-s⁢i⁢n⁢θ⁢s⁢i⁢n⁢φ+i*(s⁢i⁢n⁢θ⁢c⁢o⁢s⁢φ+c⁢o⁢s⁢θ⁢s⁢i⁢n⁢φ)]=

(8) r⁢q⁢[c⁢o⁢s⁢(θ+φ)+i*s⁢i⁢n⁢(θ+φ)]

On this foundation, we consider the modal lengths of the eigenvalues and eigenvectors that correspond precisely to the magnitude of the change in a stretch, which makes more physical sense than merely considering the real part.

Immediately following, we provide the specific modal analytical method. First, the eigenvalues are sorted by mode length from largest to smallest ${\text{Λ}}_{i}^{2}\left(i=1,\ldots ,N\right)$ and the corresponding eigenvectors are rearranged. In order to depict the actual global level of slow oscillations in the brain, the first eigenvector corresponding to the largest eigenvalue modal length was not divided at the first level. The global slow oscillations can be expressed as:


(9) $$H_{i\,n}=\frac{H_{1}}{N}=\frac{{\text{Λ}}_{1}^{2}\,M_{1}\,\left(1-p_{1}\right)}{N^{2}}$$

where the parameters and variations can be explained later.

Level 2 eigenvector separated brain regions into three sub-modules: balance, strong oscillation, and weak oscillation. After the entire module was divided into three parts, the latter oscillatory deformation was much higher than the former. (*AVG* + *SD*, *AVG* + 2_*_SD) represents a balanced oscillatory deformation while cognitive patterns are switched more flexibly, and cognition is at a greater level (complex planes ② and ⑤ in Figure 1). (*AVG* + 2_*_SD, + ∞) represent a strong oscillatory deformation with significant feature differences and shifts. Moreover, the greater the amplitude of the oscillation, the more it may negatively impair cognition (complex planes ③ and ⑥ in Figure 1). (0,*AVG* + *SD*) denotes a weakening of the oscillation level and a slowing of cognition, tending toward a mode that occurs when the oscillation gets suppressed (complex planes ① and ④ in Figure 1). Where *AVG* and *SD*represent the set’s mean and standard deviation, respectively, this set is made up of the modal lengths of all components of the whole eigenvector. Nonetheless, it is challenging to quantify the degree of rotation in measures of complex numbers. In this part, we do not distinguish between the effect of the rotation angle on the magnitude of oscillations at each level; further explanation will be given in the Section “4. Discussion.”

As the sequence of functional patterns rises, the FC network modularizes until it reaches a state of high modularity. In this process, the case from layer 2 to layer *N* represents the deformation intensity of the strong local oscillation, expressed as


(10) $$H_{s\,e}=\sum_{i=2}^{N}\frac{H_{i}}{N}=\sum_{i=2}^{N}\frac{{\text{Λ}}_{i}^{2}\,M_{i}\,\left(1-p_{i}\right)}{N^{2}}$$

where *M*_*i*_ is the number of modules within layer *i*, which is weighted as $H_{i}=\frac{{\text{Λ}}_{i}^{2}\,M_{i}}{N}\left(i=1,\ldots ,N\right)$. *N* is the number of regions in total. Nevertheless, the heterogeneous structure of module sizes causes variations in the evaluation of separation and integration parameters; hence, similar to Wang et al. (2021), we also introduce the correction factor $p_{i}=\sum_{j}\left|m_{j}-\frac{N}{M_{i}}\right|/N$, where *m*_*j*_(*j* = 1,…,*M*_*i*_) is the module size.

In addition, we set *H*_*sb*_ = *H*_*in*_−*H*_*se*_: if *H*_*sb*_ < 0, the brain’s functional network is biased toward a state of strong local oscillatory deformation, which has a facilitative effect; if *H*_*sb*_ > 0, which implies that the network is biased toward a state under which slow global oscillations cooperate, the structure of the entire network is stable; if *H*_*sb*_ = 0, a sub-stable balance is reached between strong local oscillations and slow global oscillations, and the contribution of the original network to strong local oscillations is roughly equal to the suppression of strong local oscillations by slow global oscillations. Every *H*_*sb*_ value is normalized to the range [−1, 1]. The closer *H*_*sb*_ is to –1, the greater the strong local oscillations and the more excited the proto-network state. The closer *H*_*sb*_ is to 1, the higher the degree of slow global oscillation and the greater the suppression of strong local oscillations.

**FIGURE 2:**
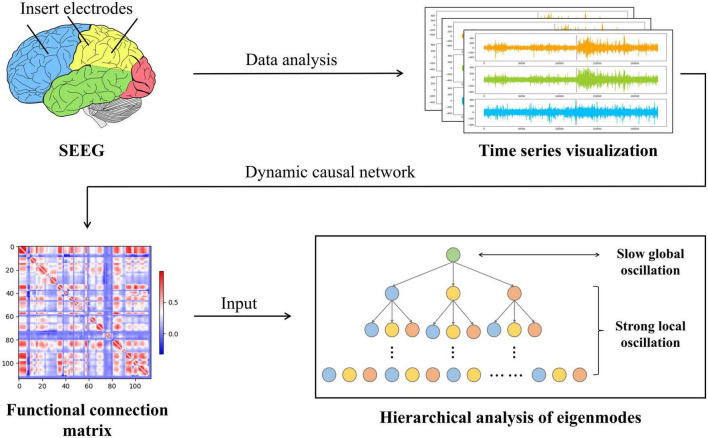
The entire workflow of the experimental procedure is in this paper. First, the SEEG data are examined, processed, and computed to establish the network of causal effects. The epilepsy causal impact network then generates a functional connectivity matrix that reflects the synchronization and causality between SEEG data from various locations. Immediately after this, we conducted a statistical analysis of the functional connectivity matrix using eigenmode hierarchical analysis to capture the slow global oscillation index vs. the strong local oscillation index for each patient under each data sample.

In conclusion, causality and synchronization were used to assess the strength of directional propagation of information flow between two epilepsy network nodes to construct a weighted epilepsy effective network. Meanwhile, we fed SEEG into this network and analyzed the output functional connection matrix. Using a modular hierarchical analysis of the eigenmodes, a dynamic description of the brain network’s strong local oscillations versus slow global oscillations in the temporal and spatial dimensions of the patient’s seizures were constructed. Ultimately, the dynamic reconfiguration of network oscillations may be the source of cognitive impairment produced by epilepsy. Figure 2 shows the main flowchart of the whole research.

## 3. Results

### 3.1. Potential reason for cognitive impairment: Disruption of the balance between strong local and slow global oscillations

**FIGURE 3:**
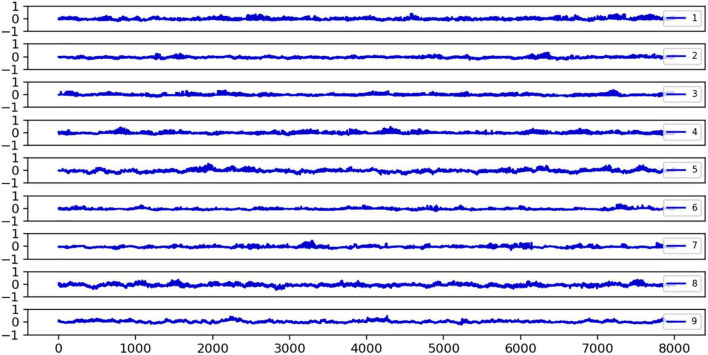
The fluctuations of the *H*_*sb*_ index over time for nine healthy subjects are plotted. *H*_*sb*_ refers to the sub-steady-state equilibrium evaluation index of slow global oscillations relative to strong local oscillations. If *H*_*sb*_ = 0, the two are in sub-steady state equilibrium; if *H*_*sb*_ < 0, the network state is more skewed toward strong local oscillations; and if *H*_*sb*_ > 0, slow global oscillations are more robust in the network. Independent of time, it was noted that the distribution of *H*_*sb*_ in healthy participants centered around 0. Each subplot’s legend numbers reflect the number of healthy subjects, the horizontal axis is the time series *t*, and the vertical axis is the *H*_*sb*_ size, normalized to the interval [−1, 1].

We discovered that epileptic seizures damage the metastable balance between local strong and slow global oscillations in large-scale networks. Specifically, we investigated the *H*_*sb*_ metrics of healthy individuals, which reflect the metastable balance between strong local oscillations and slow global oscillations. Figure 3 shows the fluctuation of the *H*_*sb*_ indication for nine healthy controls over time. We can observe that the *H*_*sb*_ fluctuates evenly around 0 and is primarily stable around 0. This indicates that healthy brain networks have balanced oscillatory states and switch between strong local oscillations and slow global oscillations more frequently, hence maintaining regular cognitive functional activity. The brain network’s facilitation of strong local oscillations plays off against the suppression of strong local oscillations by slow global oscillations, yet the network is in balance overall. For comparison, we analyzed the variation of brain network states in epileptic patients.

**FIGURE 4:**
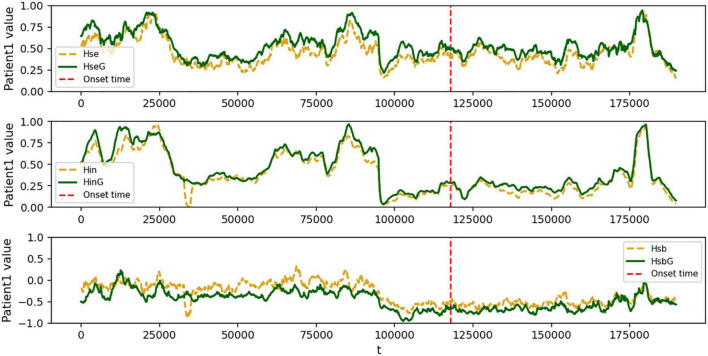
Fitted curves of the change in strong local oscillations versus global slow oscillation metrics for patient 1. In each subplot, the green (yellow) curve represents the global (local) epilepsy network, the red dashed line represents the onset time, the horizontal axis represents the time series *t*, and the vertical axis represents the *H*_*sb*_ size, normalized to [−1, 1]. Dramatic swings vary from those of healthy people and are modified by time. **(top)** the variation of patient 1’s local strong oscillations *H*_*se*_ over time; **(middle)** the change of patient 1’s global slow oscillation index *H*_*in*_ over time; and **(bottom)** the variation of patient 1’s sub-stable equilibrium index *H*_*sb*_ between strong local and slow global oscillations over time.

Patient 1 had the exact opposite features compared to healthy controls, as shown in Figure 4. The strong local oscillation index *H*_*se*_ and the slow global oscillation index *H*_*in*_ exhibited rapid, irregular fluctuations within the range [−1, 1]. Similarly, the balance index *H*_*sb*_ exhibited the same alterations (Figure 4), which seemed very distinctive from healthy individuals. The primary data implies that the disruptive impact of seizures on the metastable balance of strong local oscillations and slow global oscillations is rather severe. In addition, both strong local oscillations and slow global oscillations in the functional network were enhanced after a period of seizure. In particular, the degree of enhancement is much greater for strong local oscillations than for slow global oscillations, and it recovers to pre-seizure levels after a seizure. As a result, changing strong local oscillations and slow global oscillations in the functional network may be a pathogenic mechanism that leads to cognitive impairment. In contrast, the interictal phase is associated with a decrease in the flexibility of both strong local oscillations and slow global oscillations. We assume that not only is there cognitive impairment during seizures but also a general divergence between the configuration of the patient’s network’s functional structure and the course of cognitive activity in healthy individuals.

### 3.2. New network oscillation dynamic reconfiguration discoveries may be relevant to cognitive impairment pathophysiology

**FIGURE 5:**
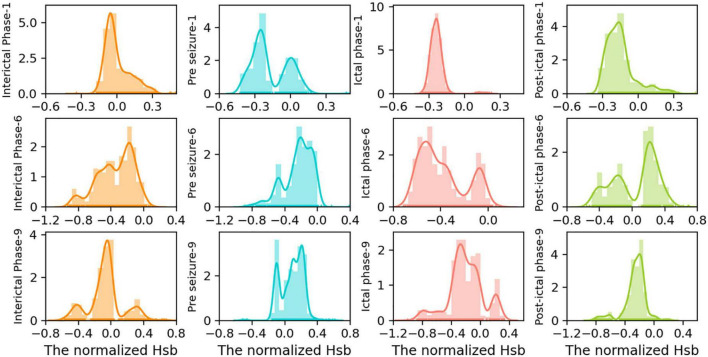
Distribution of *H*_*sb*_ kernel density of the large-scale network in patients 1, 6, and 9 at different periods of seizures. To further examine the distribution of peak density changes, we intercepted four segments with the same data points from the time series of *H*_*sb*_, indicating interictal, pre-ictal, seizure, and post-ictal phases, corresponding to the four colors from left to right in the figure. kernel density charts for various simultaneous seizure periods for patients 1, patient 6, and patient 9. In each subplot, the x-axis indicates the magnitude of the normalized *H*_*sb*_ value, the y-axis represents the density magnitude, and the y-axis labels reflect a time for a particular patient.

*H*_*sb*_ changes in the form of the distribution of peak nuclear density may be a sign of cognitive impairment caused by seizures. To further validate our conclusion, we investigated the dynamics of strong local oscillations against slow global oscillations throughout various seizure phases for each patient. Figure 5 displays the seizure periods in the *H*_*sb*_ kernel density distribution results for each of the three patients. This study demonstrates a high degree of similarity across state transitions, showing that the dynamic hierarchy of oscillatory reorganization of brain networks varies considerably. The states are divided into four groups corresponding to Figure 5’s four colors.

During the interictal phase (Figure 5, first column), the peaks were single-peaked or equally distributed, showing normal cognitive function and the ability to transition between local strong and slow global oscillations. During the pre-ictal phase, the distribution of strong local oscillations and slow global oscillations showed a bimodal pattern with independent peaks, suggesting reduced switching flexibility and good cognitive condition by epileptic waves (Figure 5, second column). Immediately after and most notably during the seizure period, the network was significantly biased toward strong local oscillations, exhibiting a single-peaked concentration pattern and accumulating negative values, suggesting almost complete cognitive loss of the brain during seizures (Figure 5, third column). Cognitive activity returned to normal in the late seizure phase, when strong local oscillations reverted to a bimodal pattern or uniform distribution with slow global oscillations, similar to the interictal phase (Figure 5, fourth column).

In contrast, the oscillatory patterns of the functional network are often diffused and distributed in states of strong local oscillations and weak global oscillations. Subsequently, seizures cause them to be concentrated in hyper-intense strong local oscillations states, gradually dispersed following the seizure. The increased concentration of network oscillatory patterns during seizures may be substantially responsible for the start of cognitive impairment associated with epilepsy. However, a fascinating phenomenon was discovered: this decrease in regularity was not absolute. In addition, the peak distribution of network oscillation patterns for a particular patient during a specific seizure period was random. However, seizure variability is not entirely random since the overall trend of attack change remained consistent between patients. Because of differences in seizure intensity, duration, and brain network regions, the impact of these specificities on the results is insignificant.

**FIGURE 6:**
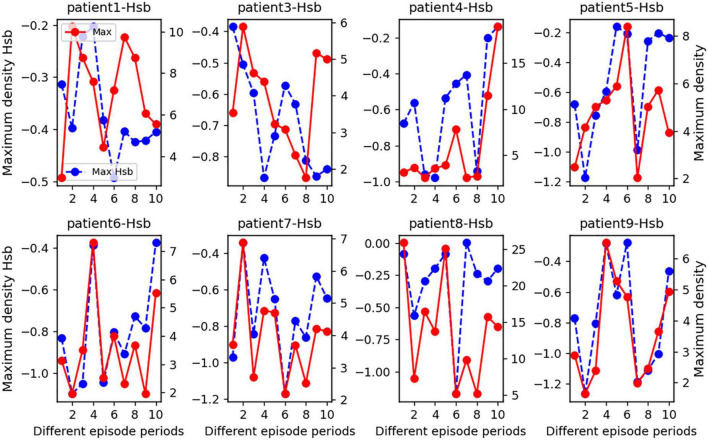
A statistical line chart of the maximal *H*_*sb*_ nucleus density for seizure durations in eight epileptic individuals. On average, we subdivided each seizure phase into two sections and chose two interictal intervals to examine. Specifically, the x-axis values of each subplot represent several seizure periods: 0–2 for the interictal period 1, 2–4 for the interictal period 2, 4–6 for the pre-ictal period, 6–8 for the seizure period, and 9–10 for the post-ictal period. In addition, we counted the peaks in the *H*_*sb*_ nucleus density distribution plot during each phase and recorded the *H*_*sb*_ values corresponding to the peaks together with the density values to create this line graph. The patient number is the title of each subplot, the blue dashed line represents the magnitude of the maximum density of *H*_*sb*_ values for each time interval, as measured by the left y-axis, and the solid red line represents the maximum density value for each time interval, as measured by the right y-axis.

In order to highlight how changes in the state of strong local oscillation and slow global oscillation vary between patient episodes, we estimated the change in the balance state between local strong and slow global oscillations at the peak for eight patients. The x-axis coordinate value of the *H*_*sb*_ distribution’s maximum density (blue dashed line in Figure 6) was drastically decreased and restored not only after a seizure but also happened many times. This analysis suggests that seizures cause a significant increase in the number of strong local oscillations in large-scale brain networks and that the inhibitory influence of slow global oscillations on strong local oscillations has almost completely disappeared at this time. However, after a seizure, protective mechanisms in the brain may prevent the loss of cognitive overload. The observed pattern of rapid increases followed by declines in concentration was commonly seen in patients, validating the generalizability of the concentration as mentioned above fluctuations. Again, based on patient-specificity, it is worthwhile to investigate that the indicated characteristics vary in time of commencement and degree of severity and may occur during either phase of the episode.

### 3.3. Pathological mechanisms that prevent cognitive loss: The oscillatory reconfiguration of networks at various sizes

Given that high levels of strong local oscillations are expected throughout a vast network of distinct patients, we can also anticipate that network features will not limit these measures. To assess this, we also identified smaller, more specialized (non-epileptogenic) local networks for each patient. According to clinical case reports, each local (non)epileptogenic network included 64 loci with (non)epileptiform discharges during the interictal and ictal phases. In addition, *H*_*sb*_ plunge intervals and the exact time and number of high-level, locally intense oscillations were evaluated.

**FIGURE 7:**
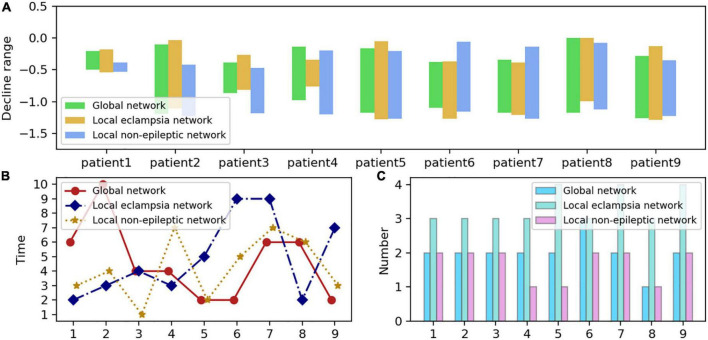
Distribution of neural network sizes in nine epilepsy patients. **(A)** The *H*_*sb*_ plunge interval for each network size is formed by the highest and lowest values of the blue curve in each subplot of Figure 6, which corresponds to the length of each bar. The y-axis value corresponding to the bottom of each bar on the graph is the blue curve, which represents the most negligible value of *H*_*sb*_, while the top represents the highest value of *H*_*sb*_. The x-axis represents the patient number, and the y-axis measures the interval maximums and minimums with the interval lengths. **(B)** Periods during which high-intensity oscillations occur in networks of various sizes, namely, the most significant decrease followed by a rebound in each subplot of Figure 6’s blue dashed line. In the graph, the three colors reflect the three network types, the x-axis represents the patient number, and the y-axis denotes the precise time during which the steepest drop occurred, numbered as in Figure 6 to represent separate seizure episodes. **(C)** The frequency of high-intensity oscillations in the various networks, mainly the frequency of the blue dash’s descent and subsequent rebound in each subplot of Figure 6. The x-axis represents the number of patients, while the y-axis displays the frequency.

Figure 7 demonstrates that despite variations in the size and type of the patient’s functional brain networks, the sudden interval of *H*_*sb*_ of each patient is the same, and the number of high-level localized strong oscillations is identical or varies by one. Notably, the sudden interval of *H*_*sb*_ was more frequent and more prolonged in the localized epileptogenic network. Thus, whereas seizures considerably impact the interplay between strong local oscillations and slow global oscillations within the localized epileptogenic network, high levels of strong local oscillations do not significantly impair cognitive performance within the brain network.

Let us consider the emergence of high levels of locally strong oscillations to represent an extreme cognitive state. It makes sense to consider the relationship between the period during which high levels of locally strong oscillations emerge and when they become clinically apparent. We discovered that the moment of creation of high-level, local, strong oscillations was random among the various patients and network types examined in this research (Figure 7). In principle, the onset of high-level strong local oscillations should correspond with the clinically characterized onset of a seizure or the number six. However, high-level local oscillations are more prevalent during the interictal or pre-ictal phase and less commonly during the late phase. Therefore, can high-level strong local oscillations be utilized as a biomarker to identify seizures, and is this information clinically relevant? Cognitive impairment brought on by high-level strong local oscillations before seizures? This needs additional investigation.

Few research has compared local non-epileptogenic networks with epileptogenic networks, another original and novel aspect of our study. First, we discovered that seizures also impact the local non-epileptogenic brain network. Although seizures are far less common in this network than in the localized epileptogenic network, this network is likely to be more intense. Second, periods with high levels of strong local oscillations in the local non-epileptogenic network were much closer to those in the global network (Figure 7). In conclusion, our findings indicate that networks of varying sizes exhibit significant levels of robust local oscillations during epileptic seizures. In response to the severe oscillatory reconfiguration of large-scale brain networks, the brain often develops pathological protective mechanisms against cognitive impairment.

## 4. Discussion

Quantitative evaluation of oscillatory reorganization is now restricted to the oscillatory frequency range. Understanding how this research significantly advanced network oscillatory recombination influences and contributed to seizure-induced cognitive impairment. The oscillatory reconfiguration of a network comprises both strong local oscillation and slow global oscillation, as well as the inhibitory and facilitative influences between them. In addition, we analyzed particular changes in oscillatory reconfiguration at various seizure periods and network limitation sizes.

Numerous studies have studied the relationship between oscillations and cognitive impairment. However, there are few theoretical investigations on the impact of network oscillations on epilepsy-induced cognitive impairment. Our experimental findings on network oscillations are consistent with earlier research. During wakefulness, the human brain reaches a critical condition and produces many transient α oscillations in global synchronization (Kim and Lee, 2020). Nevertheless, different brain disturbances (such as sleep, anesthesia, and trauma) may lead the brain to depart from the critical state (Hutt et al., 2018). This supports our hypothesis that, during the resting state, the brain is in a metastable balance between local strong and slow global oscillations and that seizures may disturb this balance. Another investigation has shown intermittent discharges contribute to cognitive impairment or epileptogenesis (Staley et al., 2005). Moreover, this investigation revealed that psychopathically elevated band θ connectivity was related to a greater incidence of seizures (Ibrahim et al., 2013). This may indicate that significantly amplified strong local oscillations may be a marker for seizures and a trigger for the onset of cognitive impairment in epileptic patients.

Our findings imply that slow global oscillations aid cognitive recovery after seizures, which is the outcome of a brain-protective mechanism. In addition, the degree of substantial enhancement of strong local oscillations was significantly greater in the local epileptogenic network than in the large-scale network, and the frequency and number of occurrences of substantial enhancement of strong local oscillations were more similar to those in the local non-epileptogenic network, which may be another manifestation of a brain protective mechanism. Slow oscillatory synchronization has been shown to contribute to functional connections across widely dispersed neuronal populations (Llinás et al., 2005). It has been discovered that abnormal slow-wave activity occurs in the brain after seizures marked by impaired consciousness (Yang et al., 2012). These findings are comparable to those we inferred for slow global oscillations.

The specificity of oscillatory recombination in various epileptic patients at different periods is of interest to us. Furthermore, several research might support our findings. For instance, the strength of network connections may be connected to individual variations in cognitive function, and spontaneous oscillatory activity may explain the diversity of task-evoked responses (Boly et al., 2008; Lewis et al., 2009). Additionally, exceptional results on local non-epileptogenic networks have been well supported by previous research. In the kainite mouse model of hippocampal epilepsy, it was shown that the rapid ripples indicative of epileptic symptoms is not localized to the lesion, despite being predominant in the lesion (Sheybani et al., 2018, 2019). Specifically, it has been shown that the time of spike appearance may connect with various cognitive impairment degrees and kinds (Fonseca et al., 2007). This also verifies the variability of the onset of substantial increases in localized slow oscillations.

It has been demonstrated that the physical process of oscillatory steady-state reverberation generates eigenvectors and eigenvalues of a system to quantify the eigenvalues implied in the geometry of external objects and their spectral representations in order to generate metrics and perform more accurate covariant inversion transformations (Jolliffe and Cadima, 2016). Moreover, similarly inside the CNS (Central Nervous System), complex eigenvalues and complex eigenvectors might indicate the contrast between covariant sensory and inverted motion vector representations of external geometry, which compose the system’s functional geometry (Pellionisz and Llinas, 1985). Our theoretical analysis then applies more practically to complex eigenvalues and complex eigenvectors. However, few studies provide a precise geometric understanding of complex eigenvalues and complex eigenvectors in high-dimensional spaces. As a result, we need to find an exact expression for the quantization of rotation angles in these spaces, which necessitates additional research.

Neurobiological systems that allow language processing are distinguished by the bidirectional flow of information in directed networks (Schoffelen et al., 2017). Graph-theoretic analysis of directed connection estimates more precisely detects the dynamic connectivity of functional networks in actual epileptic brains than undirected functional connectivity estimates (Dehaene and Changeux, 1997), creating new avenues for human connect omics. Sadly, however, current studies are more based on the role of Markov blankets on in- and out-degree, multivariate non-parametric dynamic Granger causality with directed transfer functions to build directed weighted networks (Lewis et al., 2009; Horvát et al., 2016; Zafeiriou et al., 2020). Next, graph-theoretic analysis techniques, such as typical path length, global efficiency, local efficiency, and clustering coefficients (Rentzeperis and van Leeuwen, 2020; Friston et al., 2021; Qin et al., 2022), were applied to the network. It is pleasant to observe that our analysis of directed networks utilizing the eigenmodal technique is relatively novel. However, we have yet to devote much effort to enhancing the eigenmodal technique to accurately represent the properties and dynamics of directed weighted networks. Our team will likely develop more efficient approaches and novel, generalizable findings in the future.

Nevertheless, there are still some limitations to consider when interpreting our results. For instance, we should have considered the correlation between centrality and node strength. It has been demonstrated that metrics capturing network node correlations can correctly identify motion-related regions in the pre-central and post-central gyri as critical network components and prove the expected hemispheric asymmetry (Frässle et al., 2021). Due to the private nature of the epilepsy patient data, the quantity of data in this research was inadequate to verify the generalizability of the results; hence, future attempts will focus on examining the commonality of network oscillatory reconfiguration using data from more public databases. Second, the current work lacks simulations of kinetic models to understand further the pathophysiological mechanisms behind epileptic seizures’ cognitive impairment.

The search for the underlying mechanisms of seizure-induced cognitive impairment has been a focal issue to which our study contributes to some extent. In addition, the game interaction between the network’s strong local and slow global oscillations offers a novel method to consider the above difficulties. Their combination is a significant contribution to the study of brain research, which might aid in detecting and treating brain illnesses. A deeper and more specific study of network oscillatory reorganization might be generalized to studying multiple brain disorders.

## 5. Conclusion

In conclusion, we investigate the dynamic reorganization of network oscillations, including significant enhancement of strong local oscillations during seizures, disruption of the metastable balance between local strong and slow global oscillations, and changes in the peak oscillation pattern during different periods of seizures. All of these may be potential mechanisms for cognitive impairment caused by seizures. In contrast, enhancing slow global oscillations after seizures may be a significant indicator of cognitive recovery. Significantly less dramatic than in the local epileptogenic network, the substantial enhancement of strong local oscillations in the global network occurred at periods and times more similar to those in the local non-epileptogenic network. This may be a manifestation of a brain protection mechanism. This study provides an excellent opportunity to characterize better seizure-induced cognitive impairment and its possible underlying mechanisms, which may help guide early clinical assessment and treatment aimed at preventing neuropsychological impairment in various dynamic brain function networks in epileptic patients. In addition, it offers a solid foundation for future study on weighted directed functional networks.

## Data availability statement

The original contributions presented in this study are included in the article/Supplementary material, further inquiries can be directed to the corresponding author.

## Author contributions

DF proposed and supervised the project and contributed to writing the manuscript. LQ analyzed the data, performed the experiments, and wrote the manuscript. LQ and ZY wrote the code. QW supervised the project. GL provided raw data and clinical guidance. All authors contributed to the article and approved the submitted version.

## References

[B1] Abela E. Rummel C. Hauf M. Weisstanner C. Schindler K. Wiest R. (2014). Neuroimaging of epilepsy: Lesions, networks, oscillations. *Clin Neuroradiol.* 24 5–15. 24424576 10.1007/s00062-014-0284-8

[B2] Axmacher N. Elger C. E. Fell J. (2008). Ripples in the medial temporal lobe are relevant for human memory consolidation. *Brain* 131 1806–1817. 18503077 10.1093/brain/awn103

[B3] Binnie C. D. Marston D. (1992). Cognitive correlates of interictal discharges. *Epilepsia* 33:S11–S17. 1486830

[B4] Boly M. Phillips C. Balteau E. Schnakers C. Degueldre C. Moonen G. (2008). Consciousness and cerebral baseline activity fluctuations. *Hum. Brain Mapp.* 29 868–874. 18465800 10.1002/hbm.20602 PMC6871145

[B5] Buzsáki G. (2007). The structure of consciousness. *Nature* 446 267–267. 17361165 10.1038/446267a

[B6] Buzsaki G. Draguhn A. (2004). Neuronal oscillations in cortical networks. *Science* 304 1926–1929. 15218136 10.1126/science.1099745

[B7] Dehaene S. Changeux J. P. (1997). A hierarchical neuronal network for planning behavior. *Proc Natl. Acad Sci. U.S.A.* 94 13293–13298. 10.1073/pnas.94.24.13293 9371839 PMC24302

[B8] Donner T. H. Siegel M. (2011). A framework for local cortical oscillation patterns. *Trends Cogn. Sci.* 15 191–199. 21481630 10.1016/j.tics.2011.03.007

[B9] Engel A. K. Fries P. Singer W. (2001). Dynamic predictions: Oscillations and synchrony in top–down processing. *Nat. Rev. Neurosci.* 2 704–716. 11584308 10.1038/35094565

[B10] Ewell L. A. Fischer K. B. Leibold C. Leutgeb S. Leutgeb J. K. (2019). The impact of pathological high-frequency oscillations on hippocampal network activity in rats with chronic epilepsy. *eLife* 8:e42148. 10.7554/eLife.42148 30794155 PMC6386518

[B11] Földi T. Lőrincz M. L. Berényi A. (2021). Temporally targeted interactions with pathologic oscillations as therapeutical targets in epilepsy and beyond. *Front. Neural Circ.* 15:784085. 10.3389/fncir.2021.784085 34955760 PMC8693222

[B12] Fonseca A. F. D. Herpin U. Paula A. M. D. Victória R. L. Melfi A. J. (2007). Agricultural use of treated sewage effluents: Agronomic and environmental implications and perspectives for Brazil. *Sci. Agric.* 64 194–209.

[B13] Frässle S. Manjaly Z. M. Do C. T. Kasper L. Pruessmann K. P. Stephan K. E. (2021). Whole-brain estimates of directed connectivity for human connectomics. *NeuroImage.* 225:117491. 10.1016/j.neuroimage.2020.117491 33115664

[B14] Friston K. J. Fagerholm E. D. Zarghami T. S. Parr T. Hipólito I. Magrou L. (2021). Parcels and particles: Markov blankets in the brain. *Netw. Neurosci.* 5 211–251. 10.1162/netn_a_00175 33688613 PMC7935044

[B15] Goltsev A. V. Lopes M. A. Lee K. E. Mendes J. F. F. (2013). “Critical and resonance phenomena in neural networks,” in *AIP conference proceedings*, 1510 (College Park, MD: American Institute of Physics), 28–35.

[B16] Guo J. Wu Q. Zhao C. W. Xiao B. Feng L. (2018). Dynamic functional disturbances of brain network in seizure-related cognitive outcomes. *Epilepsy Res.* 140 15–21. 10.1016/j.eplepsyres.2017.12.005 29227796

[B17] Harkin L. A. McMahon J. M. Iona X. Dibbens L. Pelekanos J. T. Zuberi S. M. (2007). The spectrum of SCN1A-related infantile epileptic encephalopathies. *Brain* 130 843–852. 17347258 10.1093/brain/awm002

[B18] Hitzer E. (2002). *Imaginary eigenvalues and complex eigenvectors explained by real geometry. In applications of geometric algebra in computer science and engineering.* Boston, MA: Birkhäuser, 145–155.

[B19] Holmes G. L. (2015). Cognitive impairment in epilepsy: The role of network abnormalities. *Epileptic Disord.* 17 101–116. 25905906 10.1684/epd.2015.0739 PMC5410366

[B20] Holmes G. L. Lenck-Santini P. P. (2006). Role of interictal epileptiform abnormalities in cognitive impairment. *Epilepsy Behav.* 8 504–515. 16540376 10.1016/j.yebeh.2005.11.014

[B21] Horvát S. Gămănuţ R. Ercsey-Ravasz M. Magrou L. Gămănuţ B. Van Essen D. C. (2016). Spatial embedding and wiring cost constrain the functional layout of the cortical network of rodents and primates. *PLoS Biol.* 14:e1002512. 10.1371/journal.pbio.1002512 27441598 PMC4956175

[B22] Hutt A. Lefebvre J. Hight D. Sleigh J. (2018). Suppression of underlying neuronal fluctuations mediates EEG slowing during general anaesthesia. *Neuroimage* 179 414–428. 10.1016/j.neuroimage.2018.06.043 29920378

[B23] Ibrahim G. M. Akiyama T. Ochi A. Otsubo H. Smith M. L. Taylor M. J. (2012). Disruption of rolandic gamma-band functional connectivity by seizures is associated with motor impairments in children with epilepsy. *PloS One.* 7:e39326. 10.1371/journal.pone.0039326 22737233 PMC3380842

[B24] Ibrahim G. M. Anderson R. Akiyama T. Ochi A. Otsubo H. Singh-Cadieux G. (2013). Neocortical pathological high-frequency oscillations are associated with frequency-dependent alterations in functional network topology. *J. Neurophysiol.* 110 2475–2483. 24004529 10.1152/jn.00034.2013

[B25] Jensen O. Kaiser J. Lachaux J. P. (2007). Human gamma-frequency oscillations associated with attention and memory. *Trends Neurosci.* 30 317–324. 17499860 10.1016/j.tins.2007.05.001

[B26] Jolliffe I. T. Cadima J. (2016). Principal component analysis: A review and recent developments. *Philos. Trans. A Math. Phys. Eng. Sci.* 374:20150202. 10.1098/rsta.2015.0202 PMC4792409 26953178

[B27] Kim M. Lee U. (2020). Alpha oscillation, criticality, and responsiveness in complex brain networks. *Netw. Neurosci.* 4 155–173. 32043048 10.1162/netn_a_00113 PMC7006877

[B28] Lévesque M. Salami P. Shiri Z. Avoli M. (2018). Interictal oscillations and focal epileptic disorders. *Eur. J. Neurosci.* 48 2915–2927. 28644911 10.1111/ejn.13628

[B29] Lewis C. M. Baldassarre A. Committeri G. Romani G. L. Corbetta M. (2009). Learning sculpts the spontaneous activity of the resting human brain. *Proc. Natl. Acad. Sci. U.S.A.* 106 17558–17563. 19805061 10.1073/pnas.0902455106 PMC2762683

[B30] Llinás R. Urbano F. J. Leznik E. Ramírez R. R. Van Marle H. J. (2005). Rhythmic and dysrhythmic thalamocortical dynamics: GABA systems and the edge effect. *Trends Neurosci.* 28 325–333. 10.1016/j.tins.2005.04.006 15927689

[B31] Pellionisz A. Llinas R. (1985). Tensor network theory of the metaorganization of functional geometries in the central nervous system. *Neuroscience* 16 245–273. 10.1016/0306-4522(85)90001-6 4080158

[B32] Qin Y. Hu Z. Chen Y. Liu J. Jiang L. Che Y. (2022). Directed brain network analysis for fatigue driving based on EEG source signals. *Entropy* 24:1093. 10.3390/e24081093 36010760 PMC9407608

[B33] Quiroga R. Q. Kreuz T. Grassberger P. (2002). Event synchronization: A simple and fast method to measure synchronicity and time delay patterns. *Physical Rev. E* 66:041904. 10.1103/PhysRevE.66.041904 12443232

[B34] Rentzeperis I. van Leeuwen C. (2020). Adaptive rewiring evolves brain-like structure in weighted networks. *Sci. Rep.* 10:6075. 10.1038/s41598-020-62204-7 32269235 PMC7142112

[B35] Sadaghiani S. Kleinschmidt A. (2016). Brain networks and α-oscillations: Structural and functional foundations of cognitive control. *Trends Cogn. Sci.* 20 805–817. 10.1016/j.tics.2016.09.004 27707588

[B36] Schalk G. McFarland D. J. Hinterberger T. Birbaumer N. Wolpaw J. R. (2004). BCI2000: A general-purpose brain-computer interface (BCI) system. *IEEE Trans. Biomed. Eng.* 51 1034–1043. 15188875 10.1109/TBME.2004.827072

[B37] Schoffelen J. M. Hultén A. Marquand A. F. Uddén J. Hagoort P. (2017). Frequency-specific directed interactions in the human brain network for language. *Proc. Natl. Acad. Sci. U.S.A.* 114 8083–8088. 10.1073/pnas.1703155114 28698376 PMC5544297

[B38] Shamshiri E. A. Tierney T. M. Centeno M. St Pier K. Pressler R. M. Sharp D. J. (2017). Interictal activity is an important contributor to abnormal intrinsic network connectivity in paediatric focal epilepsy. *Hum. Brain Mapp.* 38 221–236. 10.1002/hbm.23356 27543883 PMC6866978

[B39] Sheybani L. Birot G. Contestabile A. Seeck M. Kiss J. Z. Schaller K. (2018). Electrophysiological evidence for the development of a self-sustained large-scale epileptic network in the kainate mouse model of temporal lobe epilepsy. *J. Neurosci.* 38 3776–3791. 10.1523/JNEUROSCI.2193-17.2018 29555850 PMC6705908

[B40] Sheybani L. Van Mierlo P. Birot G. Michel C. M. Quairiaux C. (2019). Large-scale 3–5 Hz oscillation constrains the expression of neocortical fast ripples in a mouse model of mesial temporal lobe epilepsy. *Eneuro* 6:ENEURO.494–ENEURO.418. 10.1523/ENEURO.0494-18.2019 30783615 PMC6378326

[B41] Staley K. Hellier J. L. Dudek F. E. (2005). Do interictal spikes drive epileptogenesis? *Neuroscientist* 11 272–276. 16061513 10.1177/1073858405278239

[B42] Truccolo W. Ahmed O. J. Harrison M. T. Eskandar E. N. Cosgrove G. R. Madsen J. R. (2014). Neuronal ensemble synchrony during human focal seizures. *J. Neurosci.* 34 9927–9944. 25057195 10.1523/JNEUROSCI.4567-13.2014 PMC4107409

[B43] Ung H. Cazares C. Nanivadekar A. Kini L. Wagenaar J. Becker D. (2017). Interictal epileptiform activity outside the seizure onset zone impacts cognition. *Brain* 140 2157–2168. 28666338 10.1093/brain/awx143 PMC6167607

[B44] van Diessen E. Zweiphenning W. J. Jansen F. E. Stam C. J. Braun K. P. Otte W. M. (2014). Brain network organization in focal epilepsy: A systematic review and meta-analysis. *PloS One.* 9:e114606. 10.1371/journal.pone.0114606 PMC4262431 25493432

[B45] Von Stein A. Sarnthein J. (2000). Different frequencies for different scales of cortical integration: From local gamma to long range alpha/theta synchronization. *Int. J. Psychophysiol.* 38 301–313. 10.1016/s0167-8760(00)00172-0 11102669

[B46] Wang J. Deng B. Gao T. Wang J. Yi G. Wang R. (2020). Frequency-dependent response in cortical network with periodic electrical stimulation. *Chaos* 30:073130. 10.1063/5.0007006 32752642

[B47] Wang R. Liu M. Cheng X. Wu Y. Hildebrandt A. Zhou C. (2021). Segregation, integration, and balance of large-scale resting brain networks configure different cognitive abilities. *Proc. Natl. Acad. Sci. U.S.A.* 118:e2022288118. 10.1073/pnas.2022288118 34074762 PMC8201916

[B48] Watson B. O. (2018). Cognitive and physiologic impacts of the infraslow oscillation. *Front. Syst. Neurosci.* 12:44. 10.3389/fnsys.2018.00044 30386218 PMC6198276

[B49] Wilke C. Worrell G. He B. (2011). Graph analysis of epileptogenic networks in human partial epilepsy. *Epilepsia* 52 84–93. 21126244 10.1111/j.1528-1167.2010.02785.x PMC3200119

[B50] Yang Y. Hu C. Abu-Omar M. M. (2012). Conversion of glucose into furans in the presence of AlCl3 in an ethanol–water solvent system. *Bioresou. Technol.* 116 190–194. 10.1016/j.biortech.2012.03.126 22609675

[B51] Zafeiriou M. P. Bao G. Hudson J. Halder R. Blenkle A. Schreiber M. K. (2020). Developmental GABA polarity switch and neuronal plasticity in bioengineered neuronal organoids. *Nat. Commun.* 11:3791. 10.1038/s41467-020-17521-w 32728089 PMC7391775

